# Peak width of skeletonized mean diffusivity as a neuroimaging biomarker in first-episode schizophrenia

**DOI:** 10.3389/fnins.2024.1427947

**Published:** 2024-09-23

**Authors:** Man Xu, Kangkang Xue, Xueqin Song, Yong Zhang, Jingliang Cheng, Junying Cheng

**Affiliations:** ^1^Department of Magnetic Resonance Imaging, The First Affiliated Hospital of Zhengzhou University, Zhengzhou, China; ^2^Engineering Research Center of Brain Function Development and Application of Henan Province, Zhengzhou, China; ^3^Department of Psychiatry, The First Affiliated Hospital of Zhengzhou University, Zhengzhou, China

**Keywords:** schizophrenia, peak width of skeletonized mean diffusivity (PSMD), white matter integrity, diffusion tensor imaging, cognitive functions

## Abstract

**Background and objective:**

Peak width of skeletonized mean diffusivity (PSMD), a fully automated diffusion tensor imaging (DTI) biomarker of white matter (WM) microstructure damage, has been shown to be associated with cognition in various WM pathologies. However, its application in schizophrenic disease remains unexplored. This study aims to investigate PSMD along with other DTI markers in first-episode schizophrenia patients compared to healthy controls (HCs), and explore the correlations between these metrics and clinical characteristics.

**Methods:**

A total of 56 first-episode drug-naive schizophrenia patients and 64 HCs were recruited for this study. Participants underwent structural imaging and DTI, followed by comprehensive clinical assessments, including the Positive and Negative Syndrome Scale (PANSS) for patients and cognitive function tests for all participants. We calculated PSMD, peak width of skeletonized fractional anisotropy (PSFA), axial diffusivity (PSAD), radial diffusivity (PSRD) values, skeletonized average mean diffusivity (MD), average fractional anisotropy (FA), average axial diffusivity (AD), and average radial diffusivity (RD) values as well as structural network global topological parameters, and examined between-group differences in these WM metrics. Furthermore, we investigated associations between abnormal metrics and clinical characteristics.

**Results:**

Compared to HCs, patients exhibited significantly increased PSMD values (*t* = 2.467, *p* = 0.015), decreased global efficiency (*Z* = −2.188, *p* = 0.029), and increased normalized characteristic path length (lambda) (*t* = 2.270, *p* = 0.025). No significant differences were observed between the groups in the remaining metrics, including PSFA, PSAD, PSRD, average MD, FA, AD, RD, local efficiency, normalized cluster coefficient, small-worldness, assortativity, modularity, or hierarchy (*p* > 0.05). After adjusting for relevant variables, both PSMD and lambda values exhibited a significant negative correlation with reasoning and problem-solving scores (PSMD: *r* = −0.409, *p* = 0.038; lambda: *r* = −0.520, *p* = 0.006). No statistically significant correlations were observed between each PANSS score and the aforementioned metrics in the patient group (*p* > 0.05). Multivariate linear regression analysis revealed that increased PSMD (β = −0.426, *t* = −2.260, *p* = 0.034) and increased lambda (β = −0.490, *t* = −2.994, *p* = 0.007) were independently associated with decreased reasoning and problem-solving scores respectively ($R_{adj}^{2}$ = 0.295, *F* = 2.951, *p* = 0.029). But these significant correlations did not withstand FDR correction (*p_FDR* > 0.05).

**Conclusion:**

PSMD can be considered as a valuable neuroimaging biomarker that complements conventional diffusion measurements for investigating abnormalities in WM microstructural integrity and cognitive functions in schizophrenia.

## 1 Introduction

Schizophrenia is a severe psychiatric disorder characterized by positive symptoms (including hallucinations, delusions, and thought disorders), negative symptoms (including apathy, emotional flattening, and social withdrawal), and cognitive function deficits (involving working memory, social cognition, executive ability, etc.) (Sui et al., 2012; van Os et al., 2010; Marder and Cannon, 2019). Currently, there is no consensus on the pathophysiology of schizophrenia. The hypothesis of myelin dysplasia has had a profound impact, specifically, the abnormal development, differentiation and function of oligodendrocytes may be related to the pathogenesis of schizophrenia (Birur et al., 2017). Myelin consists of oligodendrocytes surrounding axons with myelinated nerve fibers primarily found in white matter (WM) (Branson, 2013; Christine et al., 2019). Schizophrenia can be considered as a disconnection syndrome resulting from abnormalities in myelin formation (Pettersson-Yeo et al., 2011).

Advancements in neuroimaging techniques have facilitated the assessment of WM structural integrity *in vivo*. Among various MRI methods, diffusion tensor imaging (DTI) is particularly well-suited for evaluating subtle WM damage (Raja et al., 2019). Specifically, quantitative parameters derived from DTI technology can reflect the abnormalities in microstructural integrity and directionality of WM tracts, consistently associated with cognition and clinical manifestation (Zanon Zotin et al., 2022). However, the widespread application of DTI in routine clinical practice is hindered by labor-intensive and time-consuming processing techniques. Recently, a fully-automated and robust imaging marker called peak width of skeletonized mean diffusivity (PSMD), calculated by the combination of “skeletonization” and histogram analysis of DTI-derived mean diffusivity (MD) images, has been proposed as an improvement over traditional DTI measures (Baykara et al., 2016). The utilization of skeletonized maps eliminates contamination from cerebrospinal fluid (CSF) while the histogram-based approach enhances the capability to characterize diffuse brain diseases (Deary et al., 2018; Low et al., 2020). PSMD reflects heterogeneity in MD values across major WM tracts and has demonstrated close associations with cognitive performance in several neurological conditions. To date, PSMD serves as a biomarker for WM pathology and cognitive manifestations primarily among patients with small vessel disease (SVD) and multiple sclerosis, yielding promising results (Deary et al., 2019; Wei et al., 2019; Raposo et al., 2021; Vinciguerra et al., 2021). A longitudinal study revealed that compared to whole brain mean diffusivity peak height and brain parenchymal fraction measurements, PSMD requires smaller sample sizes (Wei et al., 2019). Furthermore, a study on the WM of newborns revealed that, PSMD demonstrated the highest accuracy in classifying gestational age between preterm and term infants compared to other peak width of skeletonized DTI-derived coefficients (Blesa et al., 2020). Therefore, PSMD may hold great practical value for clinical research and application.

The human brain is a complex network consisting of numerous brain regions and interconnected WM axonal pathways, collectively known as the structural network connectome (Sporns, 2011). The framework of the structural connectome facilitates the investigation of the underlying widespread WM abnormalities associated with diseases (Wei et al., 2018; Cui et al., 2019). DTI-based tractography constructs curves representing maximal diffusion coherence and facilitates estimation of heterogeneous fiber bundles. Based on the identified fiber bundles, the structural connections among brain regions can be estimated to establish the WM connectome. Connectomes allow for inferences about the structural organization and integrity through graph theory analysis using parameters that reflect topological network features (He and Evans, 2010). For instance, measures of integration provide insights into the network's ability to combine information from distributed processes, while measures of segregation offer hints about the brain's capacity for parallel information processing (Fornito and Bullmore, 2015; Frey et al., 2021). Graph theory analysis can detect subtle alterations in the WM network that may be overlooked by traditional measures (Drakesmith et al., 2015).

Recent DTI researches have revealed alterations in WM integrity among individuals with schizophrenia. A comprehensive collaborative meta-analysis investigating differences in WM microstructure found that schizophrenia patients generally exhibit decreased fractional anisotropy (FA), particularly in the anterior radiation corona, corpus callosum, and genu (Kelly et al., 2018). Two studies have consistently indicated that schizophrenia patients exhibit notably elevated MD values in the superior longitudinal fasciculus (Clark et al., 2011; Waszczuk et al., 2022). Additionally, it has been reported that schizophrenia patients show significantly increased axial diffusivity (AD) and radial diffusivity (RD) values within regions of interest compared to healthy controls (HCs) (Haigh et al., 2019; Du et al., 2013; Kelly et al., 2018). With regard to the WM network of schizophrenia patients, several studies have indicated that rich club organization of connectome was significantly disrupted, the node-specific path lengths in the frontal and temporal lobe regions were significantly increased, and the global integration function was weakened (Cui et al., 2019; Heuvel et al., 2010; Wang et al., 2012; Wei et al., 2020). While findings across different studies may not be entirely consistent, they collectively support the conclusion that WM network connections in schizophrenia patients exhibit abnormalities. The relationship between WM integrity and specific neurocognitive function is significant, suggesting that WM abnormalities may contribute to cognitive dysfunction in schizophrenia (Wozniak and Lim, 2006). These findings indicate that cerebral function and structure are already altered in patients with schizophrenia compared to healthy individuals, with disruptions in WM playing a relatively critical role. However, to our knowledge, no studies have yet reported on the peak width of skeletonized diffusion metrics of WM among patients with schizophrenia.

In this study, we recruited first-episode drug-naive schizophrenia patients and HCs. Our objective was to investigate the overall microstructural integrity of WM using peak width of skeletonized diffusion metrics and conventional DTI parameters as well as structural network global topological parameters. Additionally, we aimed to assess the relationship between these metrics and clinical scales of disease. This cross-sectional study reveals that PSMD may serve as a potential neuroimaging biomarker for evaluating WM microstructural abnormalities and cognitive impairment in patients with first-episode schizophrenia.

## 2 Materials and methods

### 2.1 Subjects

This study received approval from the local ethics committee, and all participants signed written informed consent prior to undergoing MR examination. A total of 70 individuals with first-episode schizophrenia were prospectively enrolled between December 2022 and November 2023. Diagnostic evaluation was conducted by a psychiatrist using the Structural Clinical Interview for Diagnostic and Statistical Manual of Mental Disorders, Fifth Edition (DSM-V), ensuring that all patients met the diagnostic criteria for schizophrenia as outlined in DSM-V. The severity of mental symptoms in patients was quantified using the Positive and Negative Syndrome Scale (PANSS). Neurocognitive functions were assessed using the Chinese version of the MATRICS Consensus Cognitive Battery (MCCB) (Shi et al., 2015). The MCCB subtests included: (1) Trail Making Test A; (2) Brief Assessment of Cognition in Schizophrenia: Symbol coding; (3) Category Fluency Test; (4) Continuous Performance Test-Identical Pairs; (5) Spatial Span Wechsler Memory Scale; (6) Letter Number Span; (7) Hopkins Verbal Learning Test-Revised; (8) Brief Visuospatial Memory Test-Revised; (9) Neuropsychological Assessment Battery (NAB): Mazes; (10) Mayer-Salovey-Caruso Emotional Intelligence Test (Nuechterlein et al., 2008). The cognitive evaluation results were inputted into MCCB cognitive statistical software and subsequently transformed into the corresponding scores for seven domains, namely speed of processing (SOP), attention/vigilance (AV), working memory (WM), verbal learning and memory (Vrbl_Lrng), visual learning and memory (Vis_Lrng), reasoning and problem-solving (RPS), and social cognition (SC).

None of the patients received antipsychotic medication. Moreover, 65 age- and gender-matched healthy volunteers were recruited as HCs during the same period. Inclusion criteria for all subjects included right-handedness and an age range of 15 to 40 years. The duration of the disease was <3 years, with an intermission of <6 months. General exclusion criteria encompassed MRI contraindications, central nervous system disease, pregnancy, head trauma, histories of substance abuse or systemic medical diseases, and electroconvulsive therapy records. Additional exclusion criteria for HCs comprised a history of any Axis I disorder in the DSM-V and having a first-degree relative with a mental disorder. Demographic information and medical history were systematically collected for each participant. All subjects underwent a standardized neurocognitive test battery as previously described. Following image quality inspection, 14 patients and 1 HC with severe motion artifacts in MR images were excluded, thus, this study ultimately included 56 patients and 64 HCs.

### 2.2 MRI data acquisition

The participants' images were acquired using a 3.0T MRI system (Discovery MR750, GE Healthcare) equipped with an 8-channel head coil. Foam padding was applied during scanning to minimize head movement, and earplugs were provided to reduce noise interference. A sagittal 3D T1-weighted dataset was obtained through a spoiled gradient echo BRAVO sequence with the following parameters: repetition time (TR) = 8.2 ms; echo time (TE) = 3.2 ms; inversion time = 450 ms; flip angle = 12°; field of view (FOV) = 256 mm × 256 mm; acquisition matrix = 256 × 256; slice thickness = 1.0 mm; number of slices = 188. Diffusion tensor images were acquired using a single-shot spin-echo EPI sequence with the following parameters: TR = 7,100 ms; TE = 60.5 ms; FOV = 256 mm × 256 mm; acquisition matrix = 128 × 128; slice thickness = 2.0 mm; number of slices = 70; diffusion weighting in 64 directions with b-value of 1,000 s/mm^2^, along with 10 b_0_ images without diffusion weighting. Conventional MR images, including T2-weighted fluid-attenuated inversion recovery sequence and T2-weighted fast spin echo sequence, were also obtained for the purpose of excluding brain structural abnormalities and cerebrovascular diseases.

### 2.3 PSMD and DTI metrics processing

**Figure 1:**
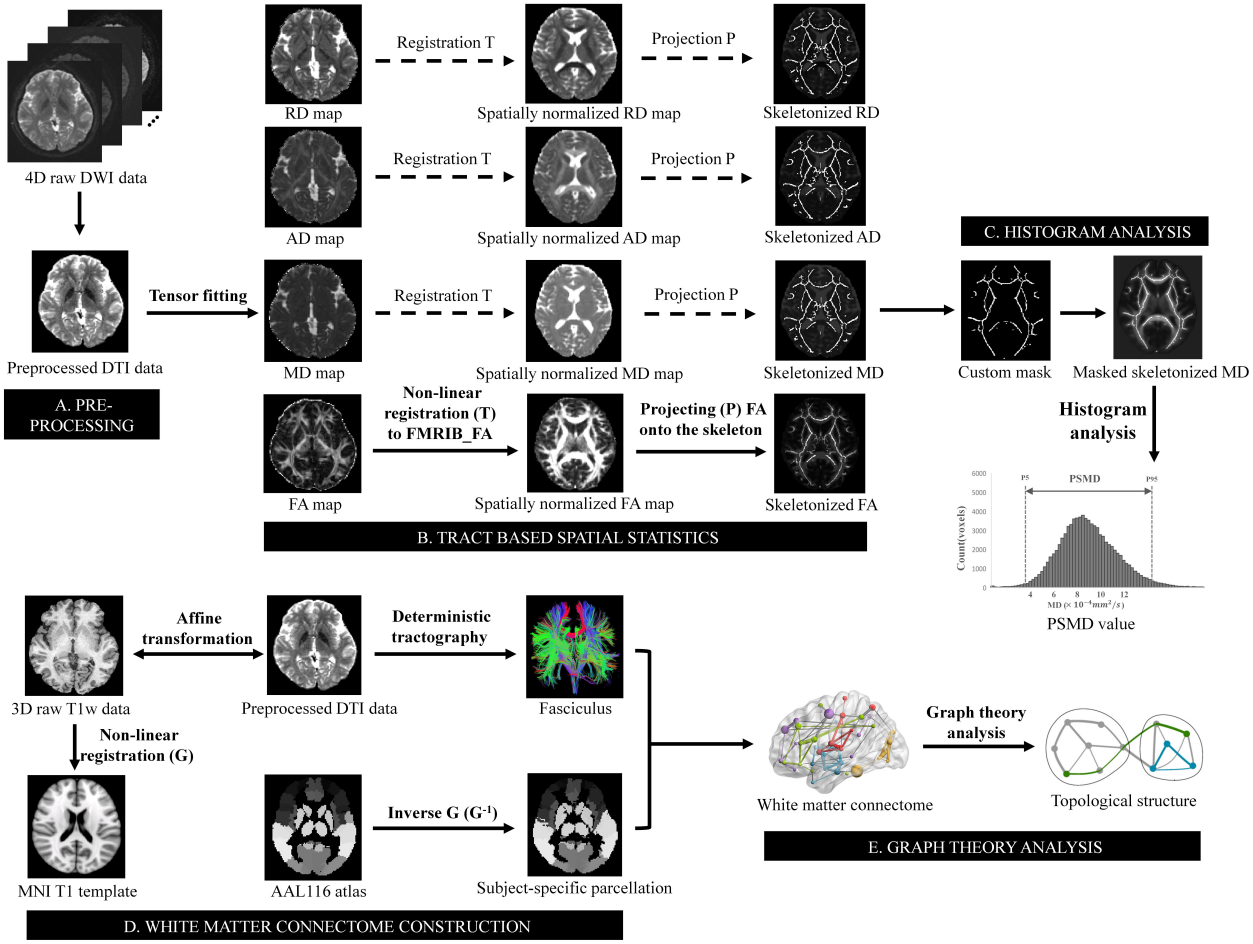
Schematic overview of the pipeline for PSMD value and topological metrics analysis. **(A)** Preprocessing steps included susceptibility, motion and eddy currents correction, brain extraction, and tensor fitting. **(B)** Skeletonization was performed using tract-based spatial statistics by non-linear registering the FA map to FMRIB FA template and projecting it onto the skeleton derived from the mean of all registered FA images. The transformation matrices also used for MD, AD, and RD maps to obtain a skeletonized map. **(C)** The skeletonized MD map was further masked using a custom-made mask. PSMD value was calculated as difference between 95th and 5th percentiles of voxel-based MD values within masked skeletonized MD map. **(D)** Non-linear registration was conducted on T1 weighted data after affine transformation to preprocessed DTI data, aligning it with T1 template in MNI space resulting in a non-linear transformation. Inverse transformation was then applied to AAL116 template for subject-specific parcellation in native space, which combined fasciculus estimated by deterministic tractography to construct structural white matter connectome. **(E)** Graph theory analysis was subsequently performed to estimate topological metrics of the connectome. PSMD, peak width of skeletonized mean diffusivity; FA, fractional anisotropy; MD, mean diffusivity; AD, axial diffusivity; RD, radial diffusivity; FMRIB, functional MRI of the brain; MNI, Montreal Neurological Institute; AAL, automated anatomical labeling.

DTI data analysis was utilized tools from the FMRIB Software Library (FSL, https://fsl.fmrib.ox.ac.uk/fsl/) (Jenkinson et al., 2012). After conversion from DICOM format to NIfTI format with dcm2niix (https://www.nitrc.org/projects/dcm2nii/) (Li et al., 2016), the raw DTI data underwent initial correction for susceptibility-induced distortions using topup (Andersson et al., 2003), followed by correction for eddy currents and head motion using eddy (Andersson and Sotiropoulos, 2016). Subsequently, brain tissues were extracted using BET (Smith, 2002), and finally entered into the tensor fitting program to obtain FA, MD, AD, and RD images using dtifit. PSMD was automatically calculated across the brain via a shell script without user intervention (available at https://www.psmd-marker.com). The script included processing steps such as skeletonization of WM tracts with Tract Based Spatial Statistics (TBSS) (Smith et al., 2006) and diffusion histogram analysis (Baykara et al., 2016). Firstly, all participants' FA images were non-linearly registered to the standard space FMRIB 1-mm FA template using the non-linear registration tool FNIRT (FMRIB Non-linear Image Registration Tool) (Andersson et al., 2007). A WM skeleton was created from the mean of all registered FA images by searching for maximum FA values in directions perpendicular to local tract direction in the mean FA image. Each subject's FA map was then projected onto this skeleton, creating individual skeletonized FA image that could be used for voxel-wise statistics. Secondly, the MD image was projected onto the mean FA skeleton to generate the MD skeleton, utilizing projection parameters derived from each participant's individualized registration process. To avoid contamination of CSF partial volume effects on the WM skeleton, MD skeletons were further masked with a standard skeleton at an FA threshold value of 0.3 and a custom mask provided within the PSMD toolbox to exclude regions adjacent to ventricles. Finally, PSMD value was calculated as difference between 95th and 5th percentiles of voxel-based MD values within WM skeleton (McCreary et al., 2020). Similarly, by employing the aforementioned processing flow in the FA/AD/RD images, the peak width of skeletonized fractional anisotropy (PSFA), axial diffusivity (PSAD), radial diffusivity (PSRD) values was obtained respectively. Besides, skeletonized average MD, FA, AD, and RD values were calculated from the corresponding skeletonized maps as the conventional DTI metrics (Figure 1).

### 2.4 Connectome topological metrics processing

The Pipeline for Analyzing Brain Diffusion Images toolkit (PANDA, https://www.nitrc.org/projects/panda) (Cui et al., 2013) was utilized to process the DTI images. For network construction, the brain parcellation framework in PANDA was employed to segment the entire brain into 116 regions of interest. Specifically, the structural T1-weighted image of each subject was initially coregistered to the corresponding non-diffusion image in the DTI native space using an affine transformation. Subsequently, the registered structural image was non-linearly transformed in the ICBM152 T1 template in Montreal Neurological Institute space. Finally, the automated anatomical labeling (AAL) 116 atlas was wrapped from standard space to each native space by inversing the non-linear transformation from previous step and defining each region as one node (Gong et al., 2009). The whole-brain WM fiber bundles were constructed using Fiber Assignment by Continuous Tracking (FACT) algorithm. Streamline terminated if fibers turned at an angle >45° or encountered a voxel with FA <0.2 due to low likelihood of belonging to bundle of interest under these conditions. The fiber number (FN) between a pair of nodes was defined as the inter-regional structural connectivity. Structural brain network was ultimately established through a subject-specific 116 × 116 FN-weighted matrix. Moreover, we utilized the brain connectivity toolbox (BCT) (Rubinov and Sporns, 2010) to process the acquired networks, involving computation and normalization of graph theoretic metrics. In this study, we extracted several global topological metrics including global efficiency (Eg), local efficiency (Eloc), normalized cluster coefficient (gamma), normalized characteristic path length (lambda), small-worldness (sigma), assortativity, modularity, and hierarchy (Sporns, 2013) (Figure 1). The total intracranial volume (TIV) for each participant was derived from T1-weighted structural images using the Computational Anatomy Toolbox (CAT 12, http://dbm.neuro.uni-jena.de/cat12/).

### 2.5 Statistical analysis

The statistical analyses were conducted using IBM SPSS software version 22. A *p* value of <0.05 was considered statistically significant. The independent two-sample *t*-test and the Chi-square test were performed to assess the inter-group differences in age, gender, education level, and TIV, respectively. Normality of data was examined with the Kolmogorov-Smirnov (KS) test to determine appropriate parametric and non-parametric tests. Continuous variables were presented as mean ± standard deviation (SD) or median (first quartile, third quartile), depending on their distribution characteristics. Comparisons between the two groups for these metrics involved either independent two-sample *t*-tests (for parametric tests) or Mann-Whitney U-tests (for non-parametric tests). The receiver operating characteristic (ROC) curves were utilized to quantitatively evaluate the discriminative capacity of peak width of skeletonized diffusion metrics and conventional diffusion markers in distinguishing patients with first-episode schizophrenia from HCs. The Delong test was employed for comparing the area under the ROC curves (AUC). To investigate associations with clinical scales, univariate and multivariate correlation analysis models adjusted for age, gender, education level, and TIV were employed to examine the relationships between PANSS scores, cognitive scores, and metrics demonstrating significant inter-group differences, respectively. In univariate analysis, partial correlation coefficient was employed. In multivariate analysis, the multivariate linear regression model, specifically hierarchical regression model, was utilized to mitigate the impact of covariates. The false discovery rate (FDR) method was applied to adjust for multiple comparisons in the correlation analyses. Results from the regression models were reported as beta (β) coefficients with statistical values. The adjusted coefficient of variation ($R_{adj}^{2}$) was used to assess the adequacy of the statistical model's fit. The presence of multicollinearity among predictors can confound estimates of individual predictor weights, therefore, variance inflation factors (VIFs) were calculated to assess collinearity between independent variables.

## 3 Results

### 3.1 Demographic and clinical characteristics

A total of 56 first-episode schizophrenia patients and 64 HCs were included in this study. The demographic characteristics, cognitive scores for all participants, and PANSS scores for the first-episode schizophrenia patients are summarized in Table 1. There were no significant differences observed between the patient and HC groups in terms of age (*t* = 1.036, *p* = 0.302), gender (χ^2^ = 0.003, *p* = 0.956), educational level (*t* = 1.324, *p* = 0.287), or TIV (*t* = 0.214, *p* = 0.831). However, the patient group exhibited significantly lower cognitive scores across all cognitive domains compared to the HC group (*p* < 0.05).

**Table 1:** Comparison of demographic and clinical characteristics between first-episode schizophrenia patients and healthy controls.

**Demographic and clinical variable**	**FES (*n =* 56) (Mean ±SD)**	**HC (*n =* 64) (Mean ±SD)**	**t/χ^2^**	***P***
Age (years)	21.48 ± 6.68	20.41 ± 4.61	1.036	0.302
Gender (male/female)	23/33	26/38	0.003	0.956
Education (years)	10.56 ± 2.69	11.64 ± 2.90	1.324	0.287
TIV (*cm*^3^)	1,568.79 ± 135.86	1,563.42 ± 138.33	0.214	0.831
**MCCB**				
SOP	28.52 ± 12.59	40.42 ± 10.05	**−6.41**	**<0.001** ^*^
AV	31.48 ± 13.75	46.58 ± 12.24	**−7.68**	**<0.001** ^*^
WM	40.55 ± 9.68	46.10 ± 6.71	**−3.12**	**0.003** ^*^
Vrbl_Lrng	34.52 ± 8.34	41.07 ± 10.34	**−4.20**	**<0.001** ^*^
Vis_Lrng	39.83 ± 11.79	44.67 ± 9.99	**−3.02**	**0.003** ^*^
RPS	33.93 ± 9.27	36.68 ± 7.12	**−2.66**	**0.008** ^*^
SC	40.52 ± 17.92	43.77 ± 9.47	**−2.12**	**0.025** ^*^
**PANSS**				
Positive	23.51 ± 5.13	/	/	/
Negative	22.55 ± 6.01	/	/	/
General	41.16 ± 8.05	/	/	/
Total score	85.66 ± 19.24	/	/	/

FES, first-episode schizophrenia; HC, healthy control; SD, standard deviation; TIV, total intracranial volume; MCCB, MATRICS Consensus Cognitive Battery; SOP, speed of processing; AV, attention/vigilance; WM, working memory; Vrbl_Lrng, verbal learning and memory; Vis_Lrng, visual learning and memory; RPS, reasoning and problem-solving; SC, social cognition; PANSS, Positive and Negative Syndrome Scale; ^*^P < 0.05 was considered significant. Bold, statistical and *P*-values were considered significant.

### 3.2 Comparison of DTI and topological metrics between groups

The DTI and topological metrics of the first-episode schizophrenia patients and HCs are summarized in Table 2. In comparison to HCs, a statistically significant increase in PSMD values was observed in the patient group (*t* = 2.467, *p* = 0.015). However, no significant differences were found between the two groups regarding PSFA, PSAD, PSRD, skeletonized average MD, average FA, average AD, or average RD values (*p* > 0.05). With respect to topological metrics, the patient group exhibited significantly decreased Eg values (*Z* = −2.188, *p* = 0.029) and increased lambda values (*t* = 2.270, *p* = 0.025) compared to those in HCs. No significant differences were observed between the groups for the remaining metrics. The results of the ROC curve analysis are summarized in Table 3. The AUC for PSMD (AUC = 0.705, 95% CI = 0.602–0.808) exceeds that of the other peak width of skeletonized diffusion metrics (PSFA/PSAD/PSRD: AUC = 0.564—0.647) and the skeletonized average diffusion coefficients (skeletonized average MD/FA/AD/RD: AUC = 0.527—0.646). Furthermore, PSMD's performance is comparable to Eg (AUC = 0.716, 95% CI = 0.615–0.817) and lambda (AUC = 0.720, 95% CI = 0.620–0.820), as evidenced by *p* values from the Delong test between PSMD and these two metrics at 0.882 and 0.838 respectively; however, it outperforms those of the remaining topological metrics (AUC = 0.540—0.680).

**Table 2:** Comparison of DTI and topological metrics between first-episode schizophrenia patients and healthy controls.

**Metric**	**FES (*n =* 56)**	**HC (*n =* 64)**	***t/Z***	***P***
PSMD (× 10^−4^*mm*^2^/*s*)	1.76 ± 0.18	1.67 ± 0.12	**2.467**	**0.015** ^*^
PSFA	0.44 ± 0.01	0.44 ± 0.01	−0.846	0.399
PSAD (× 10^−4^*mm*^2^/*s*)	7.28 ± 0.38	7.27 ± 0.35	0.104	0.917
PSRD (× 10^−4^*mm*^2^/*s*)	3.11 ± 0.16	3.08 ± 0.17	0.901	0.369
Average MD (× 10^−4^*mm*^2^/*s*)	7.82 ± 0.21	7.79 ± 0.18	0.718	0.474
Average FA	0.48 ± 0.02	0.49 ± 0.01	−1.387	0.168
Average AD (× 10^−4^*mm*^2^/*s*)	12.48 ± 0.23	12.49 ± 0.20	−0.357	0.722
Average RD (× 10^−4^*mm*^2^/*s*)	5.49 ± 0.24	5.45 ± 0.19	1.134	0.259
Eg	12.07 (11.11, 12.88)	12.76 ± 1.82	**−2.188**	**0.029** ^*^
Eloc	18.67 (16.81, 20.39)	19.48 ± 2.75	−1.136	0.256
Gamma	4.11 ± 0.36	4.04 ± 0.36	0.990	0.324
Lambda	1.20 ± 0.05	1.18 ± 0.05	**2.270**	**0.025** ^*^
Sigma	3.42 ± 0.30	3.34 (3.24, 3.56)	−0.626	0.531
Assortativity	0.12 ± 0.06	0.12 ± 0.06	0.037	0.970
Modularity	0.60 ± 0.02	0.59 ± 0.02	1.308	0.193
Hierarchy	−0.29 ± 0.06	−0.28 ± 0.07	0.460	0.646

Data were presented as the mean ± SD or median (first quartile, third quartile). FES, first-episode schizophrenia; HC, healthy control; PSMD, peak width of skeletonized mean diffusivity; PSFA, peak width of skeletonized fractional anisotropy; PSAD, peak width of skeletonized axial diffusivity; PSRD, peak width of skeletonized radial diffusivity; MD, mean diffusivity; FA, fractional anisotropy; AD, axial diffusivity; RD, radial diffusivity; Eg, global efficiency; Eloc, local efficiency; Gamma, normalized cluster coefficient; Lambda, normalized characteristic path length; Sigma, small-worldness; ^*^P < 0.05 was considered significant. Bold, statistical and *P*-values were considered significant.

**Table 3:** Results of the ROC curve analysis in DTI and topological metrics.

**Metric**	**AUC**	***P***	**95% CI**
PSMD	**0.705**	**0.047** ^*^	0.602–0.808
PSFA	0.564	0.497	0.460–0.668
PSAD	0.619	0.721	0.513–0.725
PSRD	0.647	0.380	0.543–0.750
Average MD	0.617	0.748	0.512–0.722
Average FA	0.527	0.168	0.423–0.630
Average AD	0.558	0.424	0.452–0.663
Average RD	0.646	0.388	0.542–0.750
Eg	**0.716**	**0.029** ^*^	0.615–0.817
Eloc	0.540	0.256	0.436–0.643
Gamma	0.680	0.132	0.576–0.784
Lambda	**0.720**	**0.023** ^*^	0.620–0.820
Sigma	0.633	0.531	0.528–0.738
Assortativity	0.602	0.975	0.498–0.706
Modularity	0.667	0.209	0.564–0.770
Hierarchy	0.603	0.958	0.498–0.707

ROC, receiver operating characteristic; AUC, area under the curve; CI, confidence interval; PSMD, peak width of skeletonized mean diffusivity; PSFA, peak width of skeletonized fractional anisotropy; PSAD, peak width of skeletonized axial diffusivity; PSRD, peak width of skeletonized radial diffusivity; MD, mean diffusivity; FA, fractional anisotropy; AD, axial diffusivity; RD, radial diffusivity; Eg, global efficiency; Eloc, local efficiency; Gamma, normalized cluster coefficient; Lambda, normalized characteristic path length; Sigma, small-worldness; ^*^P < 0.05 was considered significant. Bold, AUC and *P*-values were considered significant.

### 3.3 Correlational analysis between metrics and clinical scale scores

The results of the correlational analysis between clinical scale scores and the aforementioned abnormal metrics (PSMD, Eg, and lambda) are presented in Table 4. In the first-episode schizophrenia patient group, after adjusting for age, gender, education level and TIV, it was found that both PSMD values, and lambda values exhibited a significant negative correlation with reasoning and problem-solving scores (PSMD: *r* = −0.409, *p* = 0.038; lambda: *r* = −0.520, *p* = 0.006). However, following FDR correction for the *p* values, these two significant findings did not withstand multiple comparison correction (*p_FDR* > 0.05). No significant correlation was found between other cognitive functions and the aforementioned abnormal metrics, nor between PANSS scores and the metrics (*p* > 0.05).

**Table 4:** Results of correlational analysis between abnormal metrics and clinical characteristics in first-episode schizophrenia patients.

**Clinical scale**	**PSMD**			**Eg**			**Lambda**		
	***r***	***P***	***P_FDR***	***r***	***P***	***P_FDR***	***r***	***P***	***P_FDR***
**MCCB**									
SOP	−0.340	0.090	0.330	−0.169	0.409	0.807	−0.263	0.195	0.536
AV	−0.005	0.980	0.980	0.173	0.397	0.807	−0.089	0.667	0.869
WM	0.084	0.685	0.980	0.006	0.976	0.979	−0.030	0.884	0.884
Vrbl_Lrng	0.393	0.047	0.258	0.158	0.440	0.807	−0.166	0.418	0.869
Vis_Lrng	−0.018	0.932	0.980	0.012	0.954	0.979	−0.349	0.080	0.293
RPS	**−0.409**	**0.038** ^*^	0.258	0.035	0.866	0.979	**−0.520**	**0.006** ^*^	0.066
SC	0.084	0.683	0.980	0.165	0.421	0.807	0.139	0.499	0.869
**PANSS**									
Positive	0.026	0.871	0.980	0.085	0.598	0.865	0.291	0.065	0.283
Negative	−0.033	0.838	0.980	−0.149	0.352	0.807	0.059	0.715	0.869
General	0.074	0.647	0.980	−0.178	0.265	0.807	0.067	0.676	0.869
Total score	−0.035	0.828	0.980	−0.078	0.629	0.865	−0.043	0.790	0.869

*r*, correlation coefficient; PSMD, peak width of skeletonized mean diffusivity; Eg, global efficiency; Lambda, normalized characteristic path length; MCCB, MATRICS Consensus Cognitive Battery; SOP, speed of processing; AV, attention/vigilance; WM, working memory; Vrbl_Lrng, verbal learning and memory; Vis_Lrng, visual learning and memory; RPS, reasoning and problem-solving; SC, social cognition; PANSS, Positive and Negative Syndrome Scale; ^*^P < 0.05 was considered significant; FDR, false discovery rate. Bold, correlation and *P*-values were considered significant.

### 3.4 Multivariate linear regression

To investigate the potential of the metrics with inter-group differences as independent imaging markers for clinical manifestations, multivariate linear regression analysis was conducted after adjusting for age, gender, educational level and TIV. PSMD, Eg, and lambda were included as independent variables, while cognitive function score in each domain and each PANSS score served as dependent variable. In this study, all VIFs were below 2, indicating no significant multicollinearity exist. The regression analysis revealed that the constructed model had statistical significance when the reasoning and problem-solving score was considered as dependent variable ($R_{adj}^{2}$ = 0.295, *F* = 2.951, *p* = 0.029). An increase in PSMD was independently associated with a decrease in reasoning and problem-solving score (β = −0.426, *t* = −2.260, *p* = 0.034), an increase in lambda was independently associated with a decrease in reasoning and problem-solving score (β = −0.490, *t* = −2.994, *p* = 0.007), while Eg showed no significant effect on reasoning and problem-solving score (*p* > 0.05) (Table 5). However, this significant multivariate linear correlation did not withstand FDR correction (*p_FDR* > 0.05).

**Table 5:** Results of multivariate linear regression between abnormal metrics and clinical characteristics in first-episode schizophrenia patients.

**Clinical scale**	**PSMD**			**Eg**			**Lambda**			** $R^{2_{adj}}$ ^*a*^**	***F^*a*^***	***P^*a*^***	***P_FDR^*a*^***
	β	***t***	***P***	β	***t***	***P***	β	***t***	***P***				
**MCCB**													
SOP	−0.413	−1.922	0.068	−0.313	−1.464	0.157	−0.264	−1.419	0.170	0.086	1.436	0.246	0.677
AV	0.035	0.159	0.875	0.175	0.787	0.440	−0.060	−0.310	0.759	0.013	1.060	0.416	0.677
WM	0.104	0.419	0.680	0.020	0.081	0.936	−0.037	−0.171	0.866	−0.216	0.170	0.982	0.982
Vrbl_Lrng	0.512	2.391	0.026	0.242	1.136	0.268	−0.178	−0.956	0.349	0.090	1.459	0.238	0.677
Vis_Lrng	0.014	0.059	0.953	−0.045	−0.199	0.844	−0.349	−1.760	0.092	−0.033	0.851	0.545	0.749
RPS	**−0.426**	**−2.260**	**0.034** ^*^	−0.127	−0.676	0.506	**−0.490**	**−2.994**	**0.007** ^*^	**0.295**	**2.951**	**0.029** ^*^	0.319
SC	0.124	0.508	0.616	0.240	0.990	0.333	0.161	0.764	0.453	−0.178	0.296	0.932	0.982
**PANSS**													
Positive	**–**0.015	−0.092	0.927	0.101	0.700	0.488	0.291	1.911	0.064	0.131	2.080	0.079	0.435
Negative	−0.032	−0.191	0.850	−0.135	−0.880	0.385	0.050	0.310	0.758	0.022	1.161	0.348	0.677
General	0.083	0.482	0.633	−0.174	−1.106	0.276	0.045	0.269	0.789	−0.037	0.745	0.617	0.754
Total score	−0.037	−0.218	0.829	−0.068	−0.440	0.662	0.041	0.249	0.805	0.002	1.014	0.431	0.677

β, standardized beta coefficient; ^*a*^, model statistics; $R_{adj}^{2}$, adjusted R squared value; PSMD, peak width of skeletonized mean diffusivity; Eg, global efficiency; Lambda, normalized characteristic path length; MCCB, MATRICS Consensus Cognitive Battery; SOP, speed of processing; AV, attention/vigilance; WM, working memory; Vrbl_Lrng, verbal learning and memory; Vis_Lrng, visual learning and memory; RPS, reasoning and problem-solving; SC, social cognition; PANSS, Positive and Negative Syndrome Scale; ^*^P < 0.05 was considered significant; FDR, false discovery rate. Bold, statistical and *P*-values were considered significant.

## 4 Discussion

To the best of our knowledge, this study represents the first investigation into PSMD values in patients with first-episode schizophrenia. In this pilot study, we utilized peak width of skeletonized diffusion metrics and conventional diffusion metrics alongside connectome global topological metrics as neuroimaging markers to evaluate alterations in the microstructural integrity of the entire WM in first-episode schizophrenia patients, while also investigating the clinical relevance of these markers. Our findings revealed significantly higher PSMD value and normalized characteristic path length in the patient group compared to controls, while global network efficiency was significantly lower than that observed in controls. Although no significant correlations were found between PSMD values or normalized characteristic path length and PANSS scores, they exhibited a moderate-to-close correlation with the cognitive domain of reasoning and problem-solving.

Histogram analysis is a sensitive and robust method for quantifying pathological changes, as it captures the distribution of diffusivity values across the entire brain (Tofts et al., 2003). Previous studies have demonstrated that measures of histogram peak height are associated with cognitive function and can effectively characterize disease burden in small vessel diseases (Tuladhar et al., 2015; Wei et al., 2019). PSMD is a promising DTI metric that leverages the advantages of integrating diffusion histogram analysis with skeletonization of WM tracts. Calculation of this metric is fully automated, rapid, and robust, meeting the requirements for application to large sample sizes. A key characteristic of PSMD is its restriction to voxels within the WM skeleton, efficiently excluding areas more susceptible to CSF contamination and enhancing statistical power (Baykara et al., 2016). The range between the 95th and 5th percentiles on the histogram curve can provide a dispersion tendency statistic that captures diverse sources of heterogeneity in diffusion parameters across the WM skeleton, making it more sensitive to regional variability in MD (Beaudet et al., 2020). Since the identical skeletonization procedure and custom mask are applied to all individuals, the resulting histograms are inherently normalized; specifically, they are derived from an equivalent number of voxels and have comparable bin widths. This facilitates direct comparison of PSMD values among patients with varying brain volumes and contributes to explaining the consistency of PSMD values across diverse populations (Zanon Zotin et al., 2023). Consequently, compared to traditional histogram measures, PSMD demonstrates superiority in terms of processing speed, inter-scanner reproducibility, and sample size estimations.

In this study involving drug-naive first-episode schizophrenia patients, a significant increase in PSMD was observed while no significant changes were detected in PSFA, PSAD, PSRD, average MD, FA, AD, or RD values. The results of ROC curve analysis also suggest a larger AUC value for PSMD, providing support for the hypothesis that PSMD exhibits heightened sensitivity to schizophrenia-related abnormalities in WM fiber tracts. The finding that PSMD demonstrates superior discriminative ability for WM microstructural abnormalities compared to other peak width of skeletonized diffusion coefficients is consistent with the conclusion drawn by Blesa et al. (2020), who attribute this difference to variations in inter-group variability between the 5th and 95th percentiles of the histogram. Previous studies on schizophrenia and SVD disease have demonstrated correlations between MD and myelin density, tissue rarefaction, axon count, and WM microinfarcts (van Veluw et al., 2019; Lee et al., 2013). It is plausible that similar histopathologic alterations are associated with PSMD, reflecting disruption of connectivity and deceleration of synaptic transmission, thereby contributing to cognitive impairment (Zanon Zotin et al., 2023). Higher PSMD values were found to be associated with extensive damage to structural connections and suggested diffuse damage to myelin content and axon counts in the brain of patients, possibly indicating decreased fiber integrity and pathological aggregation of free water in the extracellular compartment (Vinciguerra et al., 2021). The result highlights the potential value of using PSMD as an additional neuroimaging metric beyond conventional DTI measures during early stages of schizophrenia.

The topological alterations of the WM networks between the patients and HCs were further investigated using deterministic tractography and connectivity-based analysis methods. The WM networks in the patient group exhibited typical small-world properties, indicating that the small-world network can tolerate disease-related structural alteration to some extent (He et al., 2009). Small-worldness represents a balance between segregation, which refers to distributed and specialized processing in specific brain regions, and integration, which refers to the capacity to integrate information from distribution processes (Frey et al., 2021). However, compared with HCs, the patient group demonstrated significantly decreased global efficiency and significantly increased shortest path length. Global efficiency relies on intact long-distance connections as they facilitate communication among remote brain regions (Watts and Strogatz, 1998). The reduced global efficiency indicated a decline in effective interaction and neural information transmission across distant cortical areas. This finding aligns with previous schizophrenia fMRI studies reporting insufficient effective integration among distributed functional cortical regions (Kambeitz et al., 2016; Wang et al., 2024). Additionally, the increased shortest path length signifies prolonged optimal interactions among neurons that are crucial for functional cognitive processes within and across the brain regions. The degeneration of fiber tracts is generally considered as a potential cause for the increase in shortest path length in the patient group. The observed alterations of WM network topology suggest that there is a decrease in patients' brain capacity to effectively integrate distributed information.

The present study revealed that among all cognitive domains, only the scores in the reasoning and problem-solving domain exhibited a significant independent negative correlation with PSMD and lambda values. Although the negative correlation loses statistical significance after correction, we still regard it as a correlated trend worth discussing. Reasoning and problem-solving function was assessed using the NAB maze tracking task, which involves inductive reasoning—a crucial aspect of generating predictions and one of the most significant problem-solving activities (Wass et al., 2012). In terms of the relationship between reasoning and problem-solving function and diffusion indicators, Zahr et al. (2009) discovered a positive correlation between problem-solving function and FA values in genu and fornix. The discrepancy in the correlation between fiber bundle FA from aforementioned study and PSMD reported in this paper, regarding the reasoning and problem-solving scores, provides convergent validity for the biological meaningfulness of tracked fibers' integrity (Zahr et al., 2009). The observed correlations support the notion that degradation of WM fiber integrity serves as a biological source of related functional compromise, potentially limiting neural systems' capability to compensate for impaired function. Additionally, it has been suggested that the test employed to assess the reasoning and problem-solving domain is speed-based (Mohn and Rund, 2016). The increased lambda value observed in this study indicates prolonged optimal interactions among neurons—essential for functional cognitive processes within brain regions as well as across them—may result in decreased processing speed during cognitive tasks (Sporns, 2011).

Our study has certain limitations. Firstly, the relatively small sample size of our cohort may account for the lack of significance in cognitive correlates after applying multiple comparison correction. Therefore, our findings should be considered as preliminary and require external validation in larger cohorts. Secondly, the cross-sectional design of our study restricts causal interpretation of results and emphasizes the necessity for larger longitudinal studies. Thirdly, the FACT streamlining algorithm utilized for network construction has inherent limitations in tracking fibers within complex white matter architecture, particularly in cases of crossing fibers. A better alternative method is to obtain the advanced white matter imaging techniques such as high angular resolution diffusion imaging (HARDI) and diffusion spectrum imaging (DSI), which can yield superior qualitative data pertaining to multiple crossing fibers with high spatial orientation. Lastly, although PSMD benefits a single global measurement, its limitations lie in the absence of anatomical information which limits specificity in identifying underlying pathological abnormalities. Future investigation should incorporate comprehensive analyses to confirm the efficacy of PSMD in terms of sensitivity to microstructural changes over time and response to treatment.

In summary, this study investigated abnormalities in DTI metrics and network topological metrics in individuals with first-episode drug-naive schizophrenia, and introduced PSMD as a diffusion indicator for schizophrenia for the first time. Our findings revealed that PSMD was able to detect WM microstructural integrity abnormalities that were not identified by conventional diffusion metrics. Furthermore, PSMD demonstrated consistency with the network topological metric lambda in detecting correlations with reasoning and problem-solving cognitive dysfunction. These results suggest that PSMD is an effective neuroimaging biomarker for schizophrenia, which can complement conventional diffusion measurements to investigate cognitive impairment. Importantly, the evaluation of PSMD was rapid and fully automated, while its robustness and accuracy have been previously validated across multi-center datasets. Therefore, PSMD holds promise as a valuable neuroimaging biomarker for schizophrenia in future studies aiming to supplement quantification of WM microstructural lesions or retrospectively analyze existing DTI datasets.

## Data Availability

The raw data supporting the conclusions of this article will be made available by the authors, without undue reservation.
